# Are reframing strategies more effective than empathy in processing trauma reports? A pilot study

**DOI:** 10.3389/fpsyg.2023.1150475

**Published:** 2023-07-03

**Authors:** Sophie Leuteritz, Stephanie Böhme, Andreas Mühlberger, Werner Greve

**Affiliations:** ^1^Department of Clinical Psychology and Psychotherapy, University of Regensburg, Regensburg, Germany; ^2^Department of Clinical Psychology and Psychotherapy, University of Erlangen Nuremberg, Erlangen, Bavaria, Germany; ^3^Institute for Psychology, University of Hildesheim, Hildesheim, Lower Saxony, Germany

**Keywords:** empathy, secondary trauma, reframing, positive reappraisal, objective distancing, anxiety, depression

## Abstract

Listening to trauma reports can lead to the development of symptoms associated with secondary traumatization. This is particularly relevant for psychotherapists in practice, where psychologists need to estabilish effective strategies for processing and coping with such emotionally challenging events. This explorative study investigated adaptive reframing strategies for future therapists listening to trauma stories compared to feeling empathy for the client. In a mixed design, 42 postgraduate psychology students were randomly instructed to objectively distance themselves, reappraise, or feel empathetic while watching a video of a presumed trauma patient reporting a single violent act. An overall ANOVA did not reveal a difference between the reframing groups and the empathy group (between subjects manipulated) in their skin conductance level and heart rate variability during the video, as well as their change in state depression and state anxiety over the three measurements (before the video, after the video, and 2 days later). Nevertheless, an explorative *t*-test showed a significantly weaker rise in state depression and state anxiety from before the video to after the video in the reframing groups compared to the empathy group. This supports the suggestion that reframing strategies can be discussed as a protective factor against health issues such as secondary traumatization in therapists and should be examined in further studies in more detail.

## Objective

Empathy is considered an important resource in almost all psychotherapeutic treatment approaches (e.g., Burns and Nolen-Hoeksema, 1992; Schechter and Goldblatt, 2011). Across very different traditions, the ability (and willingness) to empathize with patients' emotional states is considered an important condition for psychotherapeutic success (e.g., Elliott et al., 2011). Even beyond therapeutic interactions and relationships, empathy is a positive resource for how people interact with each other in professional and personal relationships. Thus, it has been discussed as a supportive condition of, perhaps even a basis for, prosocial behavior (e.g., Bierhoff, 2002), and many authors have attempted to define, differentiate, and subdivide the construct (for an overview, see Elliott et al., 2011). However, feeling empathy is not without a downside for psychotherapists, especially emotional empathy, defined as the ability to feel what someone else feels (Shamay-Tsoory et al., 2008). Emotional empathy differs from cognitive perspective-taking. This difference can be observed in the structure of the human brain (Shamay-Tsoory et al., 2008; Banissy et al., 2012). Emotional empathy develops very early in life “via the process of emotional contagion” (Hatfield et al., 2009, p. 1). However, much later, we learn to distinguish others' emotions from our own (Singer and Lamm, 2009).

In the context of the phenomenon of “secondary traumatization,” authors have pointed out that emotionally empathic co-experiencing of suffering and severe distress experienced by others can also be distressing for the empathic person (e.g., Figley, 1995). Currently, there is a body of evidence indicating that individuals who have not been directly affected by trauma but who have heard vivid accounts from trauma victims or have close relationships with trauma victims may themselves exhibit reactions (symptoms) similar to those of trauma victims (Figley, 1995; Hensel et al., 2015). It is plausible to speculate that this effect is partly mediated by empathic co-experiencing of the stresses experienced “secondarily” (Figley, 1995; Daniels, 2007). Evidence suggests that the emotional component of empathy, which means attempting to feel what the patient feels, enhances the risk of secondary traumatization (Thomsen et al., 2017). Therefore, therapeutic work with patients who report traumatic experiences should put therapists at risk for secondary traumatization precisely because empathy is often considered an important aspect of the therapeutic stance for increased therapeutic success (e.g., Elliott et al., 2011). Daniels (2006) reported that every third trauma therapist had experienced symptoms like hyperarousal, intrusions, nightmares, and emotional blunting.

However, since the majority of “trauma” psychotherapists are not affected by secondary traumatization, even when confronted with highly intense reports of stressful experiences, there appear to be resources that buffer the impact of stressful reports. Numerous studies have pointed out helpful strategies in this context that are negatively associated with secondary traumatization, including social support (Ortlepp and Friedman, 2002; Galek et al., 2011), self-care (Bober and Regehr, 2006; Akinsulure-Smith et al., 2012), and problem-focused coping (Dunkley and Whelan, 2006; Gil and Weinberg, 2015).

The present study assumes that individual processes and emotion regulation strategies could play an important role in preventing secondary trauma symptoms. Such processes have been widely studied in relation to basic emotions (Gross and Levenson, 1995; Gross, 1998) and mental disorders (Martin and Dahlen, 2005; Garnefski and Kraaij, 2006). However, there has been a notable absence of investigations concerning their role in preventing secondary traumatization. This study aims to examine the (short-term) buffering role of two emotion regulation strategies in particular: positive reappraisal and objective distancing. Positive reappraisal involves reappraising a negative event, at first glance, positively (McRae et al., 2012), and it has been shown in several studies to be favorable for the emergence of positive and decreasing negative emotions (Troy et al., 2018). There is also evidence of physiological responses [e.g., a lower skin conductance level (Wolgast et al., 2011; McRae et al., 2012)]. Objective distancing, i.e., taking the perspective of an “imagined observer” or “appropriately professional” (Powers and LaBar, 2019, p. 46), is also associated with protective effects [e.g., reducing negative affect (Königsberg et al., 2010); reducing symptoms of depression (Kross and Ayduk, 2008)]. By examining the buffering role of these two emotion regulation strategies, we strive to establish guiding ideas for further studies to prevent secondary trauma in young therapists.

We hypothesized that using a reframing strategy differs from using emotional empathy in the change of negative emotions (state anxiety and state depression as an indicator of early distress) between the three measurements of our study. From before (t1) to directly after (t2) the video, where students listen to a traumatic story, the negative emotions of the emotional empathy group should rise higher than those of the reframing groups, and they should decrease less from t2 to t3. Moreover, state anxiety and state depression should increase less from t1 to t2 and decrease more from t2 to t3 in positive reappraisal than in objective distancing. We also expected a higher low-frequency/high-frequency ratio (HRV), skin conductance level (SCL), rumination, mental distress, and intrusion in the empathy group compared to the reframing groups.

## Method

### Participants

Overall, 42 psychology students (36 women and 6 men) from the University of Regensburg, who were at least attending their fourth undergraduate semester, participated in the experiment. The exclusion criteria included a diagnosed mental disorder, a cardiovascular disorder, and the intake of psychotropic drugs. None of the participants had to be excluded, and all participants completed the experimental session. Overall, four students did not participate in the follow-up measurement. One person did not mention their code, so there were 37 cases of all three measurements. The mean age was 24.62 years (*SD* = 6.16; range = 19–56).

### Procedure

Participants were welcomed in a laboratory at the University of Regensburg. They were given written information about the study, and they were asked to provide written informed consent. They generated a code and wrote their age, sex, and semester. The students then replied to a couple of questions on a computer. Among the questions were the Positive Reappraisal Scale of the CERQ (Loch et al., 2011), self-created questions about their distancing trait, the German questionnaire of empathy and perspective taking [Fragebogen für Empathie und Perspektivenübernahme (Maes et al., 1995)], and the state (t1) and trait versions of the German State-Trait-Anxiety-Depression-Inventory [STADI (Laux et al., 2013)] to control for trait influences. Afterward, three Ag/AgCl electrodes were placed on the upper body, and two Ag/AgCl electrodes were placed on the non-dominant palm. To test the effect of emotion regulation strategies in contrast to the “risk factor” empathy, we ensured that the participants were double-blind, randomized, and assigned to three groups, each given different instructions for viewing the video: emotional empathy, positive reappraisal, or objective distancing. In the emotional empathy group, the participants were instructed to feel emotionally connected to the patient and empathize with the patient's feelings. In the positive reappraisal group, the participants were instructed to reappraise the information heard positively and think of the possibility that people can emerge stronger from bad experiences. Participants in the objective distancing group were instructed to be professional and listen from a distanced perspective. After reading the instructions, the participants observed a patient reporting a traumatic experience to a therapist in a 15-min video that pretended to be an excerpt from a real trauma therapy session. The session was acted out by a psychologist from our research team and an independent actor playing a 50-year-old female patient who suffers from PTSD symptoms due to a single violent offense. In the video, the “patient” describes how she was hit by a stranger in her front yard and reports nightmares, despair, fear of leaving the house, and hyperarousal symptoms by crying and looking emotionally distressed. While watching the video, SCL and HRV were measured as indicators of physiological arousal during their exposure to the video.

**Figure 1:**
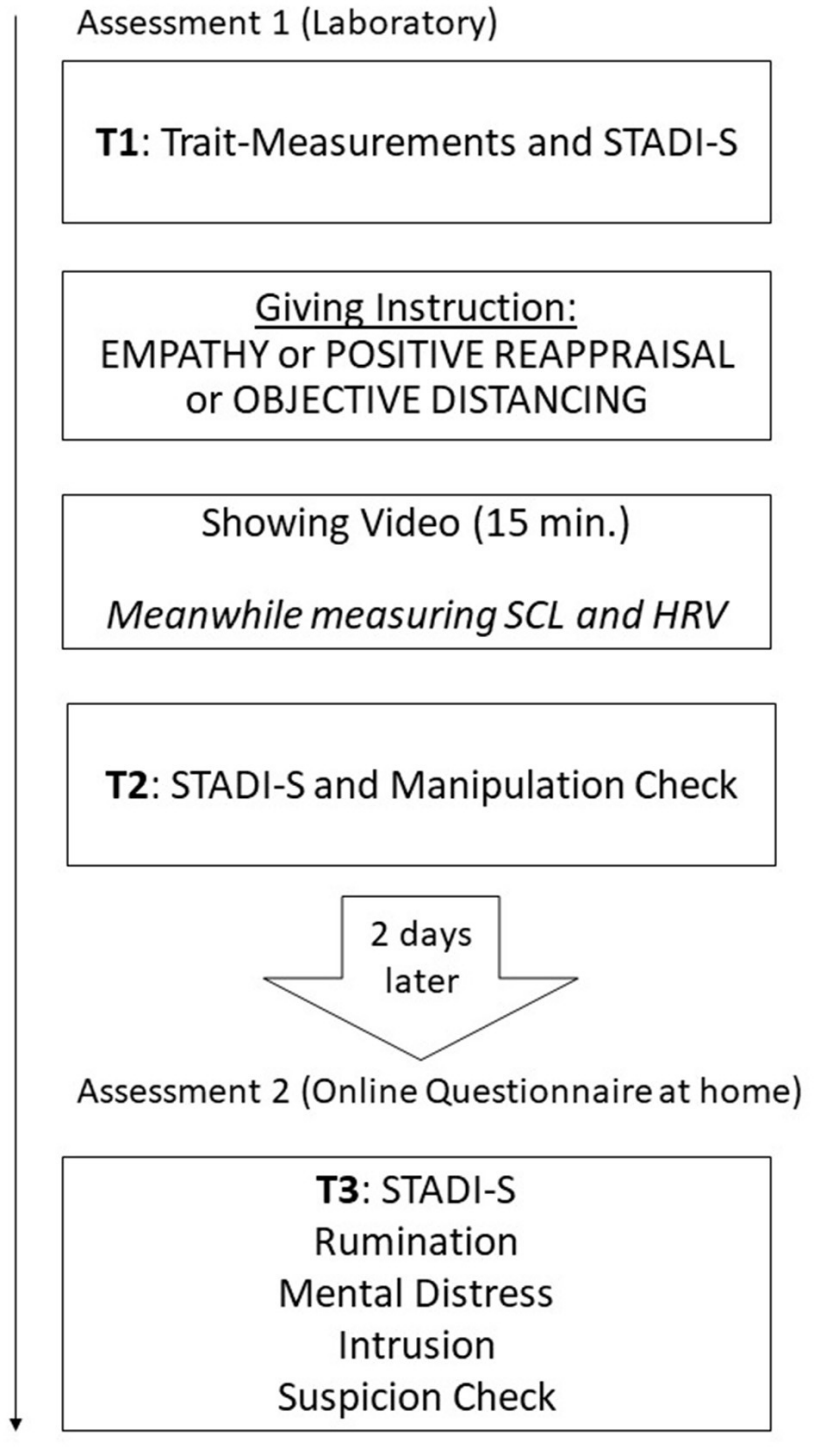
Timeline.

When the video had ended, the participants answered the STADI-S for a second time (t2), three items for a manipulation check, and 10 items about the content of the video. Two days later, the participants received a link to an online questionnaire, in which they filled in the STADI-S for a third time (t3) and answered single questions for rumination, mental distress, and intrusion. It ended with a check on whether the participants found the video “authentic.” Finally, the purpose of the study was released. See the timeline of procedure in Figure 1.

The study was approved by the Ethics Committee of the University of Regensburg.

## Assessment

### Primary outcome measurement

The State Version of the German State-Trait-Anxiety-Depression-Inventory [STADI (Laux et al., 2013)] consists of two subscales for depression (euthymia and dysthymia) and two subscales for anxiety (agitation and concern) with five items each, for example, “I'm happy” or “I'm depressed.” The 20 items are rated from 1 (not at all) to 4 (a lot). The total scale shows good reliability (t1: α = 0.82; t2: α = 0.91; t3: α = 0.89). For the measurement of mental distress, rumination, and intrusion at t3, three items of the German Questionnaire for Secondary Traumatization [Fragebogen zur Sekundaeren Traumatisierung (Daniels, 2006)] were used.

### Physiological indicators

HRV and SCL were measured by applying three electrodes on the upper body and two Ag/AgCl electrodes on the non-dominant palm. For measuring the HRV, an electrocardiogram was recorded using the Brain Vision Recorder (vAMP 16, Brain Vision, USA; sampling rate 1,000 Hz).

### Pre-processing data and statistical tests

The SCL and ECG were pre-processed using the Brain Vision Analyzer 2.1.2 (Brain Products GmbH, Munich). The SCL was high-cut filter, and segmented from the onset to the end. It was baseline corrected (1,000 ms). The mean activity was exported. The ECG was filtered with a high-cut filter at 30 Hz and a low-cut filter at 1.5 Hz. The data were then segmented from the onset to the end and baseline corrected as well. ECG data were further processed with Artifact software (Kaufmann et al., 2011): R-peaks were detected, and artifacts were semi-automatically detected and deleted. In the end, the LF/HF ratio was calculated.

Missing data in the STADI were filled in with the means of the scale, as proposed in the manual published by Laux et al. (2013). Scales were tested for distribution, outliers, and internal consistencies.

To test the main hypothesis, we used a two-factor mixed ANOVA. For the apriori contrasts, we conducted a one-way ANOVA by calculating the difference of the dependent variables between the measurements in advance and then comparing them to each other. The group difference for SCL was calculated with a one-way ANOVA and single apriori contrasts. The group difference for HRV was calculated with the non-parametric Kruskal-Wallis-H-test due to its non-normal contribution. For the single-item AVs of mental distress, rumination, and intrusion, non-parametric Mann–Whitney U-tests were used. Calculations were conducted with SPSS, an effect size calculator (Lenhard and Lenhard, 2016), and G^*^Power (Faul et al., 2007).

## Results

**Figure 2:**
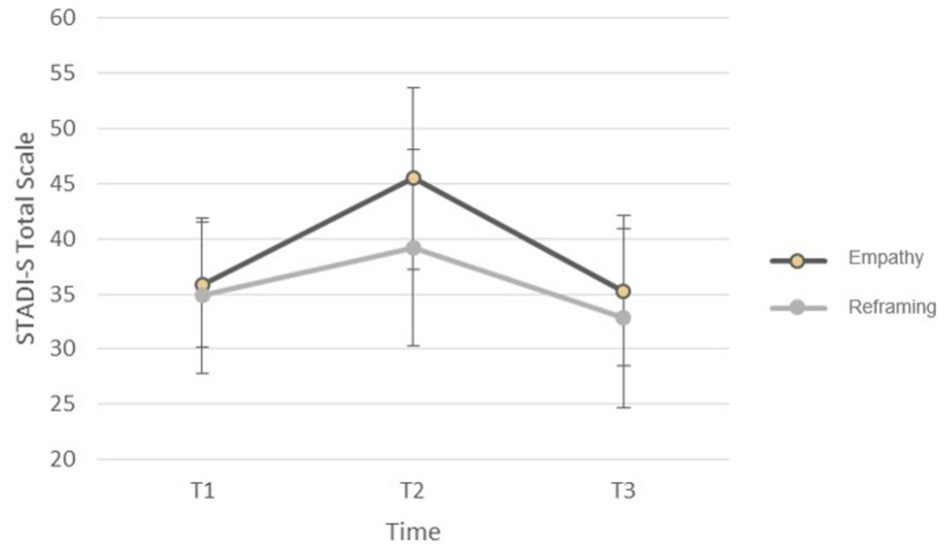
Joined state anxiety and state depression (STADI-S Total Scale) before the video (T1), after the video (T2), and 2 days later (T3) for the instruction “empathy” and the joined reframing instructions (objective distancing and positive reappraisal). Error bar, standard deviation of the mean.

A significant main effect (“time”) showed that negative emotions (state anxiety and state depression) changed in the total sample over all three measurements, *F*_(2, 35)_ = 25.46, *MSE* = 27.10, *p* < 0.001 (t1: *M* = 34.6, *SD* = 6.47; t2: *M* = 40.29, *SD* = 9.24; t3: *M* = 33.53, *SD* = 6.47). The main effect of the instruction (“group”) was not significant, *F*_(1, 35)_ = 2.25, *p* = 0.142. In particular, there was no significant interaction between the factors instructions (reframing and empathy) and time (t1, t2, and t3) for state anxiety and state depression; *F*_(2, 35)_ = 25.47, *MSE* = 27.10, *p* = 0.094, ${\eta_{p}}^{2}$ = 0.07. Thus, the instruction did not lead to a difference in negative emotion when considering all three measurements. However, specific explorative analyses on the change between the successive time points dependent on group revealed a greater increase of negative emotion between t1 and t2 in the empathy group than in the reframing groups (instruction ^*^ time), *F*_(1, 41)_ = 5.39, *MSE* = 60.92, *p* = 0.025, ${\eta_{p}}^{2}$ = 0.12 (see also Figure 2). No such difference was found between t2 and t3, *F*_(1, 35)_ = 2.37, *MSE* = 54.96, *p* = 0.133, ${\eta_{p}}^{2}$ = 0.06.

Comparing the consequences of the two reframing instructions, objective distancing and positive reappraisal, with respect to the change in state depression and state anxiety over the three measurements, there was no significant interaction of these two reframing strategies over time; *F*_(2, 42)_ = 1.73, *MSE* = 19.43, *p* = 0.191, ${\eta_{p}}^{2}$ = 0.08. Furthermore, there was no difference between these two reframing strategies when comparing the single measurements (t1 with t2: *F*_(1, 27)_ = 2.33, *MSE* = 41.43, *p* = 0.139, ${\eta_{p}}^{2}$ = 0.08; t2 with t3: *F*_(1, 21)_ = 0.38, *MSE* = 38.14, *p* = 0.546, ${\eta_{p}}^{2}$ = 0.02). The main effect “group” was not significant, *F*_(1, 21)_ = 0.97, *p* = 0.335. However, there was an overall difference between the measurements (main effect “time”), *F*_(2, 42)_ = 12.18, *p* < 0.001.

A one-way ANOVA did not reveal a difference between the groups' objective distancing, positive reappraisal, and empathy concerning the skin conductance level, *F*_(2, 39)_ = 0.29, *MSE* = 1.34, *p* = 0.749, ${\eta_{p}}^{2}$ = 0.02. A non-parametric test neither found a difference in heart rate variability between the groups of objective distancing, positive reappraisal, and empathy, $x_{\left(2\right)}^{2}$ = 1.26, *p* = 0.533, ${\eta_{p}}^{2}$ = 0.02.

U-tests of differences between reframing and empathy in secondary trauma symptoms two days after watching the video (t3) were not significant for rumination (U = 159.50, *p* = 0.959, ${\eta_{p}}^{2}$ < 0.01), mental distress (U = 149.00, *p* = 0.589, ${\eta_{p}}^{2}$ < 0.01), or intrusions (U = 143.00, *p* = 0.520, ${\eta_{p}}^{2}$ < 0.01).

Of the 38 participants, nine rated the video a little authentic — a three or less on a scale from 1 = not authentic at all to 5 = very authentic (*M* = 4.11, *SD* = 1.06). Excluding these participants did not lead to significant differences in the analyses.

## Discussion

To our knowledge, the present study is the first to examine early indicators of secondary traumatization, combining this issue of clinical counseling and therapy with emotion regulation strategies in an experimental design.

The significant main effect, “time,” indicated the general effect of the video (induction of state anxiety and state depression). In contrast to our hypothesis, there was no significant interaction between the three measurements (“time”) and the different instructions (“group”).

In an explorative analysis, we observed a significantly lower increase in negative emotions in the joint reframing groups compared to the empathy group from before the video (t1) to after the video (t2). This explorative result suggests that positive reappraisal and objective distancing could also have a short-term buffering effect on secondary trauma symptoms in counseling and therapy. This short-term adaptive effect of reframing strategies is in line with several findings of emotion regulation studies that found more positive and fewer negative emotions with the positive reappraisal instruction (Ray et al., 2008; Rood et al., 2012; Lohani and Isaacowitz, 2014; Troy et al., 2018) and the objective distancing instruction (Königsberg et al., 2010; Denny and Ochsner, 2014). In contrast to these adaptive strategies, emotional empathy can be considered a risk factor for negative emotions in therapists, as Thomsen et al. (2017) reported in their longitudinal study about secondary traumatization. Similar to the “trauma track” in the study of Speisman et al. (1964), empathy presents a maladaptive contrast to emotion regulation strategies. Nevertheless, the short-term adaptive effect on self-reported negative emotions was not detectable with respect to physiological parameters (SCL and HRV).

An important limitation of the present study is its limited power, particularly for the between-subjects ANOVAs. A *post-hoc* power analysis revealed a small power for HRV (1-ß = 0.11) and SCL (1-ß = 0.10). The small sample size resulted from the limited availability of appropriate participants (students of psychology, advanced with respect to their education, clinically interested, etc.). Hence, a replication of the present study should attempt to plan an appropriate sample size, given the relatively small effects found in the present study. Another limitation of the study is the selection of participants who are not used to listening to traumatic details. Further studies should include experienced trauma therapists to investigate whether reframing strategies can buffer secondary traumatic stress.

For this pilot study, we chose psychology students since a sufficient effect of the confrontation with traumatic details was necessary to achieve the central aim of the study (the assumed effectivity of emotion regulation processes). A high degree of experience (and expertise) in emotion regulation could have masked any reaction.

It can also be discussed that the distress might not have immediately started at the beginning of the video and that one should only compare critical segments of the video. By doing this in an explorative *post-hoc* analysis, we did not find a difference in physiological arousal between the instructions.

Examining the third point of measurement, 2 days after the participants had watched the video, the negative emotions dropped in all instructions, as it was intended for ethical reasons. The assumed greater decrease among the reframing groups compared to the empathy group could not be found. Remarkably, there was a significantly lower rise in the reframing groups to t2. However, the study conditions were relatively conservative in that the video did not lead to increased distress (negative emotions) in t3, and participants were students of a master's degree who were familiar with psychological tests. It is reassuring that the video did not induce secondary trauma.

Nevertheless, it was strong enough to make the short-term adaptive effect of the reframing strategies visible, although those were not trained in advance. Unfortunately, the study could not differentiate stress-buffering effects between the reframing strategies assessed in this study (positive reappraisal and objective distancing). The manipulation check in this study indicated that both strategies could possibly be used at the same time. Further studies should examine the specific effect mechanisms of the single strategies.

In addition, the intentional “use” of emotion regulation strategies is an empirically and theoretically open question. As a rule, emotion regulation is not a consciously governed and entirely controlled behavior. Hence, the effect of a direct instruction (which entails that it can be used as a selected means in a goal-directed action) might be relatively weak, which is why we interpreted the result of the *post-hoc t*-test with respect to the t1/t2 buffer effect (irrespective of weak effect and weak power) as a possible indicator of a buffering effect of reframing strategies. If even an emotional reaction that is just instructed (and not systematically practiced) proves to have any effect, then further systematic training can be expected to be even more effective. A follow-up step would be the investigation of individual differences in the particular emotion regulation preparedness (“competence”). This line of research might prove relevant for the importance of intentional (or non-intentional) use of reframing strategies for therapy outcomes. In particular, the question of whether objective distancing and positive reappraisal might go alongside or even balance empathy as an important factor for therapy outcome needs to be researched (e.g., Elliott et al., 2011).

## Data availability statement

The raw data supporting the conclusions of this article will be made available by the authors, without undue reservation.

## Ethics statement

The studies involving human participants were reviewed and approved by University of Regensburg Ethics Committee. The patients/participants provided their written informed consent to participate in this study.

## Author contributions

SL, SB, WG, and AM contributed to the study conception and design as well as to the material preparation. Data collection was performed by SL. Analyses were initially performed by SL, additional analyses were discussed and planned by all authors. The first draft of the manuscript was written by SL and SB. All authors commented on previous versions, contributed to the final version, and explicitly approved the final manuscript.
